# N-LODDS: A Novel Integrated Lymph Node Staging System Enhancing Prognostic Accuracy in Non-Small-Cell Lung Cancer

**DOI:** 10.1245/s10434-025-19005-x

**Published:** 2026-01-20

**Authors:** Qiying Chen, Meihong Yao, Zishan Chen, Shiwen Liu, Jinman Zhuang, Xi Chen, Jie Yi, Binghua Tu, Ziyue Yang, Yinghong Yang, Fei He

**Affiliations:** 1 Department of Epidemiology and Health Statistics, The School of Public Health, Fujian Medical University, Fuzhou, Fujian China; 2 Department of Pathology, Fujian Medical University Union Hospital, Fuzhou, Fujian China; 3 Maternal and Child Health Hospital of Fuzhou Second General Hospital, Fuzhou, Fujian China; 4 Fujian Digital Tumor Data Research Center, Fuzhou, Fujian China

**Keywords:** Lymph node metastasis, Surveillance, Epidemiology, and End Results (SEER), Non-small cell lung cancer (NSCLC), Nomogram, Log odds of positive lymph nodes (LODDS)

## Abstract

**Background:**

This study aimed to develop and validate a novel lymph node staging system integrating anatomical location and quantitative characteristics, evaluate its prognostic prediction efficacy in non-small-cell lung cancer (NSCLC), and establish a multivariate prognostic model.

**Methods:**

A total of 23,676 patients with NSCLC from the SEER database (2010–2015) were enrolled. Optimal cutoffs for lymph node parameters (NPLN, LNR, LODDS) were determined using X-tile software. Composite variables (N-NPLN, N-LNR, N-LODDS) were constructed by integrating N staging. Independent prognostic factors were screened via Cox regression, and a nomogram was developed. Performance was assessed using the receiver operating characteristic curves, calibration curves, and decision curve analysis.

**Results:**

N-LODDS staging demonstrated optimal prognostic prediction, significantly outperforming N-LNR and N-NPLN. The nomogram incorporating N-LODDS, tumor size, and nine independent prognostic factors showed superior discrimination and calibration (5 year area under the curve 0.740; 95% confidence interval 0.731–0.749) in both training and validation cohorts, with significant advantages over the TNM staging system (all *P*<0.001).

**Conclusion:**

The N-LODDS staging system significantly improves prognostic accuracy by integrating anatomical and quantitative lymph node features, providing a novel tool for personalized NSCLC management. Future multicenter prospective studies are needed to validate its clinical utility.

**Supplementary Information:**

The online version contains supplementary material available at 10.1245/s10434-025-19005-x.

## Implications for Practice

The N-LODDS staging system integrates anatomical and quantitative lymph node features, offering superior prognostication for resected N0-N2 non-small-cell lung cancer. Its core clinical implications include that it can improves the detection of occult metastasis in N0 patients, guiding adjuvant therapy; the nomogram enables individualized survival prediction, outperforming TNM staging; and it mandates standardized lymph node dissection/quantification to prevent under-staging. Prospective validation is warranted, but this provides a practical postoperative management framework.

## Introduction

According to the 2022 data released by the International Agency for Research on Cancer, global cancer-related deaths reached 9.7 million cases, and lung cancer fatalities accounted for nearly 2.5 million cases, representing one-eighth of all cancer mortality and ranking first among cancer-related deaths.^1^ Non-small-cell lung cancer (NSCLC) accounts for 85% of all lung cancer cases.^2^ Lymph node metastasis is one of the primary metastatic pathways in lung cancer and a critical factor affecting patient prognosis. As an important clinicopathological indicator for evaluating lung cancer prognosis, lymph node metastasis staging plays a crucial guiding role in formulating treatment strategies.^3,4^ The current widely adopted clinical staging standard for lung cancer is the 8th edition TNM classification system established by the American Joint Committee on Cancer (AJCC)/Union for International Cancer Control, whose lymph node (N) staging is based solely on the anatomical location of metastatic lymph nodes while disregarding quantitative factors—a limitation that may compromise staging accuracy.^5^ The 9th edition TNM staging system, recently updated, improves this classification by dividing N2 into N2a (single-station metastasis) and N2b (multi-station metastasis). Although it introduces lymph node quantity information, it still prioritizes anatomical location as the main staging criterion.^6^

Recent studies suggest that the number of metastatic lymph nodes holds significant value in nodal staging and prognostic evaluation.^5,7,8^ In esophageal cancer, for instance, early-stage classification traditionally relies on the anatomical location of primary and metastatic lesions, whereas current guidelines have incorporated lymph node quantity as a key parameter.^6,9,10^ Similarly, in breast cancer^11^ and certain gastrointestinal malignancies,^12,13^ N staging has integrated the number of metastatic lymph nodes as a reference standard. In NSCLC studies, metrics including the number of positive lymph nodes,^14,15^ lymph node ratio (LNR),^16,17^ and log odds of positive lymph nodes^18,19^ serve as independent prognostic factors with critical roles in outcome prediction. However, both N staging and other lymph node quantity-related variables reflect only a single dimension of metastatic status, and few studies have systematically explored their combined prognostic predictive efficacy or compared integrated models incorporating these parameters with conventional N staging.

In recent years, nomograms have emerged as vital predictive tools in cancer personalized medicine by translating complex prognostic models into individualized risk assessments for clinical decision-making.^20–22^ Although several internally and externally validated prognostic models exist for NSCLC,^16,18^ none integrate lymph node staging features. This study therefore proposes a novel lymph node staging integration method using the Surveillance, Epidemiology, and End Results (SEER) database and simultaneously evaluates anatomical location and quantitative characteristics of metastatic lymph nodes by combining conventional N staging with lymph node burden parameters. Based on this integrated variable, we developed a prognostic nomogram to precisely predict cancer-specific survival. This innovative staging approach enhances prognostic accuracy and provides foundational evidence for establishing new postoperative pathological staging criteria in lung cancer, ultimately optimizing clinical decision-making.

## Materials and Methods

### Data Sources and Patient Selection

**Fig. 1:**
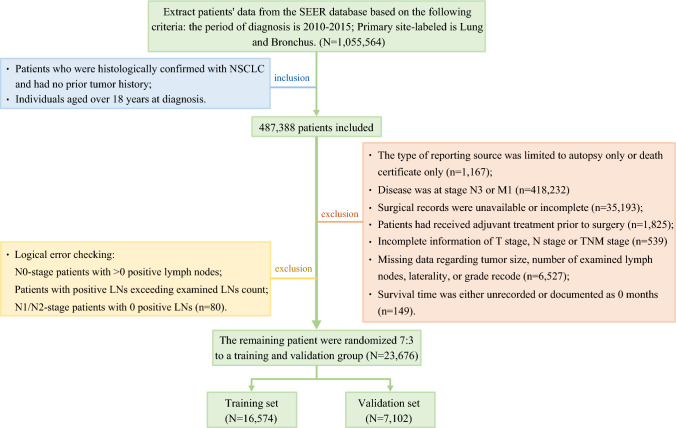
Patients’ enrollment flow diagram. LNs, lymph nodes; NSCLC, non-small-cell lung cancer; SEER, Surveillance, Epidemiology, and End Results

This study was based on SEER research data from 17 registries in the November 2023 Sub (2000–2021) in SEER*Stat version 8.4.5. Our analysis was restricted to the 2010–2015 diagnosis period to ensure staging homogeneity, driven by the fact that the SEER database provides derived AJCC 7th edition staging variables exclusively for this timeframe. Our initial cohort comprised 1,055,564 cases with a primary site of lung and bronchus identified within this timeframe. Inclusion criteria required histologic confirmation of NSCLC, absence of prior malignancy history, and age ≥18 years. Exclusion criteria included (1) inappropriate surgical intervention or recommendation of surgical treatment after neoadjuvant therapy for the late stage of NSCLC (stage N3/M1); (2) survival time <1 month; (3) neoadjuvant therapy prior to surgery; (4) missing critical clinical parameters (primary site, surgical record, tumor size, histological grade, number of lymph nodes examined, TNM stage or survival time); (5) logical inconsistencies in recorded data (N0-stage patients with >0 positive lymph nodes; patients with positive lymph nodes exceeding examined lymph node count; N1/N2-stage patients with 0 positive lymph nodes); and (6) the type of reporting source was limited to autopsy only or death certificate only. The complete screening flow diagram is presented in Fig. 1. Following screening, 23,676 eligible patients were ultimately enrolled and randomly allocated into training (*N*=16,573) and validation (*N*=7,103) sets at a 7:3 ratio for subsequent predictive modeling and analysis. This study was exempt from ethical review as all data were derived from publicly available anonymized datasets and therefore did not require approval from the institutional review board and was not subject to informed consent requirements.

### Outcomes and Variable Definition

Cancer-specific survival (CSS) was the primary endpoint of this study. Follow-up was calculated from the date of pathologic diagnosis until the occurrence of lung cancer-related death, death from other causes (follow-up endpoint ends on the date of death), or last confirmed follow-up (for patients still alive or lost to follow-up). Inclusion variables included demographic characteristics: race, age group at diagnosis (≤65 years/66–80 years/>80 years); tumor characteristics: primary site, laterality, maximum tumor diameter grouping (≤3 cm, >3–5 cm, >5–7 cm, >7 cm), tumor extension, number of regional lymph nodes detected, and number of regional lymph node metastases; histopathological features: histology (adenocarcinoma/squamous cell carcinoma/other NSCLC) and differentiation grade; treatment information: surgery of primary site and postoperative adjuvant radiotherapy or systemic therapy; and staging classification (AJCC TNM 7th edition staging).

### Calculation, Integration, and Screening of Different Lymph Node Stage

For the assessment of lymph node metastasis quantity, this study calculated two quantitative evaluation indicators based on the number of positive regional lymph nodes (NPLN) and the number of dissected lymph nodes (NDLN): lymph node ratio (LNR = NPLN/NDLN), and log odds of positive lymph nodes (LODDS = log_10_ [(NPLN+0.05)/(NDLN-NPLN+0.05)]. Optimal cutoff values for NPLN, LNR, and LODDS were determined using X-tile software version 3.6.1 (Yale University, New Haven, CT, USA). For cases with lymph node metastasis, NPLN was stratified into NPLN1 (0<NPLN≤4) and NPLN2 (NPLN >4), and LNR was categorized as LNR1 (0<LNR≤0.5) and LNR2 (LNR>0.5). LODDS stage divides all lymph node statuses into two categories; LODDS1 (≤ − 0.68) and LODDS2 (> − 0.68). These variables were integrated with N staging to create composite variables (N-NPLN, N-LNR, N-LODDS), jointly reflecting anatomical distribution and quantitative burden of nodal metastases.

Independent risk factors were screened by univariate and multivariate Cox regression (*P*<0.05). Kaplan–Meier curves were used to demonstrate the survival distribution after variable integration, and differences between subgroups were analyzed using the log-rank test (*P*-values adjusted via the Benjamini–Hochberg method). After combining subgroups that showed minimal survival differences and were not statistically significant, the N-LODDS variables with the best discriminatory power were selected for inclusion in the prognostic model based on the receiver operating characteristic (ROC) curve.

### Statistical Analysis

Categorical variables were presented as frequencies (percentages) with intergroup comparisons conducted via chi-squared tests, and continuous variables were expressed as mean ± standard deviation and analyzed using Student’s *t*-test. Prognostic factors were screened using Kaplan–Meier survival curves and univariate Cox regression (*p*<0.05). Independent predictors included in the multivariate model were sex, age group, histology, tumor size, differentiation grade, tumor extension, radiotherapy, systemic therapy, and N-LODDS. Based on multivariate Cox proportional hazards regression analysis, a nomogram for predicting CSS was constructed using the rms package. To quantify the importance of each variable in predicting CSS, the relative contribution of each predictive variable was estimated in the nomogram as the percentage of its *χ*^2^ value to the total *χ*^2^ of the model. In the training and validation sets, the performance of the model was evaluated as follows: (1) Discrimination assessment using time-dependent ROC curves with bootstrap resampling (1000 iterations) to calculate 95% confidence intervals (CIs), followed by comparative analysis against TNM stage; (2) Calibration testing was performed by plotting calibration curves (the calibration curve is used to compare predicted probabilities with observed frequencies; the closer the curve is to the 45° diagonal, the better the calibration); (3) Clinical utility quantification via decision curve analysis to determine threshold probability-dependent net benefit; and (4) Both the net reclassification improvement and integrated discrimination improvement were systematically computed to quantitatively evaluate the incremental predictive performance of the novel prognostic model against established staging benchmarks, specifically TNM stage. All statistical analyses were performed using R software (version 4.4.3). All *p*-values were two-sided, and *p*<0.05 was considered statistically significant.

## Result

### Patient Characteristics

This study included 23,676 eligible patients with NSCLC from the SEER database (2010–2015), with a median follow-up duration of 77 months (interquartile range 35–102). In the overall cohort, 52.1% were female, 83.4% were white, and 52.2% were aged 65–80 years. Primary tumor sites were classified by International Classification of Diseases for Oncology (ICD-O-3) codes, with most tumors located in the upper lobe (C34.1; 58.1%) or lower lobe (C34.3; 33.1%), and right-sided laterality was more frequent (58.1%). Adenocarcinoma (59.8%) and squamous cell carcinoma (29.5%) were the dominant histological subtypes, and grade II (45.0%) were most frequent, followed by grade III and grade IV (35.9%). In total, 61.7% of the cases had tumor diameter ≤3 cm, and the tumor expansion was mainly regional (53.3%) and localized (44.1%). Regarding treatment, 79.8% of patients underwent partial pneumonectomy, 23.1% received postoperative systemic therapy, and 7.8% underwent adjuvant radiotherapy. According to TNM stage, stage IA (39.5%) and stage IB (23.8%) were the most common diagnostic stages, and cases of stage IV were rare (0.5%). N staging revealed 78.2% as N0, 13.4% as N1, and 8.4% as N2. The total cohort was randomly divided into a training set (*n*=16,574) and a validation set (*n*=7102) at a 7:3 ratio. All baseline characteristics were balanced between the two sets (Table 1).

**Table 1:** Characteristics between the training set and the validation set

Characteristics	Total	Training set	Validation set	*P*
No. of cases	23,676	16,574	7102	
Race				0.826
White	19,749 (83.4)	13,802 (83.3)	5947 (83.7)	
Black	2013 (8.5)	1416 (8.5)	597 (8.4)	
Other	1874 (7.9)	1328 (8.0)	546 (7.7)	
Unknown	40 (0.2)	28 (0.2)	12 (0.2)	
Sex				0.818
Female	12,326 (52.1)	8620 (52.0)	3706 (52.2)	
Male	11,350 (47.9)	7954 (48.0)	3396 (47.8)	
Age group, years				0.055
≤65	9597 (40.5)	6704 (40.4)	2893 (40.7)	
>65 to ≤80	12,354 (52.2)	8703 (52.5)	3651 (51.4)	
>80	1725 (7.3)	1167 (7.0)	558 (7.9)	
Primary site				0.355
C34.1: upper lobe, lung	13,748 (58.1)	9633 (58.1)	4115 (57.9)	
C34.2: middle lobe, lung	1278 (5.4)	905 (5.5)	373 (5.3)	
C34.3: lower lobe, lung	7837 (33.1)	5463 (33.0)	2374 (33.4)	
C34.0: main bronchus	178 (0.8)	137 (0.8)	41 (0.6)	
C34.8: overlapping lesion of lung	335 (1.4)	228 (1.4)	107 (1.5)	
C34.9: lung, NOS	300 (1.3)	208 (1.3)	92 (1.3)	
Laterality				0.472
Left	9916 (41.9)	6916 (41.7)	3000 (42.2)	
Right	13,760 (58.1)	9658 (58.3)	4102 (57.8)	
Primary tumor size, cm				0.406
≤3	14,615 (61.7)	10,219 (61.7)	4396 (61.9)	
>3–5	5749 (24.3)	4029 (24.3)	1720 (24.2)	
>5–7	2133 (9.0)	1477 (8.9)	656 (9.2)	
> 7	1179 (5.0)	849 (5.1)	330 (4.6)	
Tumor extension				0.375
Regional	12,614 (53.3)	8801 (53.1)	3813 (53.7)	
Localized	10,431 (44.1)	7317 (44.1)	3114 (43.8)	
Distant	631 (2.7)	456 (2.8)	175 (2.5)	
Histology				0.243
Adenocarcinoma	14,154 (59.8)	9953 (60.1)	4201 (59.2)	
Squamous cell cancer	6974 (29.5)	4828 (29.1)	2146 (30.2)	
Other NSCLC	2548 (10.8)	1793 (10.8)	755 (10.6)	
Differentiation grade				0.304
I	4536 (19.2)	3208 (19.4)	1328 (18.7)	
II	10,652 (45.0)	7470 (45.1)	3182 (44.8)	
III & IV	8488 (35.9)	5896 (35.6)	2592 (36.5)	
Systemic therapy				0.556
No/unknown	18,212 (76.9)	12,767 (77.0)	5445 (76.7)	
Yes	5464 (23.1)	3807 (23.0)	1657 (23.3)	
Radiotherapy				0.451
No/unknown	21,825 (92.2)	15,293 (92.3)	6532 (92.0)	
Yes	1851 (7.8)	1281 (7.7)	570 (8.0)	
Surgery				0.941
Excision or resection of less than one lobe, NOS	2802 (11.8)	1973 (11.9)	829 (11.7)	
Partial pneumonectomy, NOS	3197 (13.5)	2219 (13.4)	978 (13.8)	
Lobectomy with mediastinal lymph node dissection	15,688 (66.3)	10,966 (66.2)	4722 (66.5)	
Lobe or bilobectomy extended	857 (3.6)	608 (3.7)	249 (3.5)	
Pneumonectomy	1072 (4.5)	765 (4.6)	307 (4.3)	
Extended pneumonectomy	16 (0.1)	12 (0.1)	4 (0.1)	
Extended radical pneumonectomy	13 (0.1)	9 (0.1)	4 (0.1)	
Resection of lung	6 (0.0)	5 (0.0)	1 (0.0)	
Surgery, NOS	25 (0.1)	17 (0.1)	8 (0.1)	
AJCC V7 N stage				0.342
N0	18,517 (78.2)	12,927 (78.0)	5590 (78.7)	
N1	3163 (13.4)	2249 (13.6)	914 (12.9)	
N2	1996 (8.4)	1398 (8.4)	598 (8.4)	
AJCC V7 T stage				0.336
T1	10,658 (45.0)	7412 (44.7)	3246 (45.7)	
T2	9269 (39.1)	6501 (39.2)	2768 (39.0)	
T3	2976 (12.6)	2104 (12.7)	872 (12.3)	
T4	773 (3.3)	557 (3.4)	216 (3.0)	
AJCC V7 TNM stage				0.339
IA	9360 (39.5)	6515 (39.3)	2845 (40.1)	
IB	5641 (23.8)	3961 (23.9)	1680 (23.7)	
IIA	3028 (12.8)	2121 (12.8)	907 (12.8)	
IIB	2422 (10.2)	1706 (10.3)	716 (10.1)	
IIIA	3098 (13.1)	2171 (13.1)	927 (13.1)	
IIIB	127 (0.5)	100 (0.6)	27 (0.4)	
NDLN	10.54 ± 8.49	10.61 ± 8.54	10.36 ± 8.37	0.033
NPLN	0.60 ± 1.75	0.61 ± 1.79	0.58 ± 1.65	0.149
LNR	0.06 ± 0.16	0.06 ± 0.16	0.06 ± 0.16	0.620
LODDS	− 1.04 ± 0.48	− 1.04 ± 0.48	− 1.03 ± 0.47	0.622

Patient characteristics are presented as mean ± standard deviation for continuous variables and frequency (%) for categorical variables. The differences between the training set and the validation set were assessed using Student’s *t*-test (for continuous variables) and Chi-squared test (for categorical variables)

AJCC, American Joint Committee on Cancer; NOS, not otherwise specified; NSCLC, non-small-cell lung cancer

### Integration and Selection of Lymph Node Staging Methods

Optimal cutoff values for NPLN, LNR, and LODDS were determined using X-tile software and integrated with the 7th edition *N*-stage to create new variables (N-NPLN, N-LNR, and N-LODDS). As shown in Table 2, NPLN was stratified into NPLN1 (0 < NPLN ≤4) and NPLN2 (NPLN >4), LNR into LNR1 (0 < LNR ≤0.5) and LNR2 (LNR >0.5), and LODDS into LODDS1 (≤ − 0.68) and LODDS2 (> − 0.68). Univariate and multivariate Cox regression analyses demonstrated that LODDS, NPLN, and LNR served as independent prognostic factors in quantifying lymph node metastatic burden. When integrating N staging with NPLN or LNR, all patients were stratified into five subgroups (N0-NPLN0, N1/N2-NPLN1/2, N0-LNR0, N1/N2-LNR1/2), and six subgroups were generated with LODDS (N0/N1/N2-LODDS1/2). Kaplan–Meier analysis revealed survival distribution patterns across variable-stratified groups, with Log-rank tests identifying intergroup differences in overall survival.

**Table 2:** Univariate and multivariate cox analysis of different lymph node stage methods and integrated staging systems

Variables	Description	Number (%)	1 year survival rate (95% CI)	3 year survival rate (95% CI)	5 year survival rate (95% CI)	Univariate analysis HR (95% CI)	*P*	Multivariate analysis HR (95% CI)	*P*
NPLN									
NPLN0	NPLN=0	12,927 (78.0)	0.950 (0.946–0.954)	0.862 (0.856–0.868)	0.795 (0.788–0.803)	Reference		Reference	
NPLN1	0<NPLN ≤4	3039 (18.3)	0.865 (0.853–0.877)	0.656 (0.639–0.674)	0.545 (0.527–0.564)	2.43 (2.29–2.58)	<0.001	1.64 (1.51–1.77)	<0.001
NPLN2	4<NPLN	608 (3.7)	0.810 (0.779–0.842)	0.513 (0.473–0.555)	0.387 (0.349–0.430) ^a^	3.76 (3.39–4.17)	<0.001	2.36 (2.09–2.66)	<0.001
LNR									
LNR0	LNR = 0	12,927 (78.0)	0.950 (0.946–0.954)	0.862 (0.856–0.868)	0.795 (0.788–0.803)	Reference		Reference	
LNR1	0<LNR ≤0.5	3160 (19.1)	0.869 (0.857–0.881)	0.661 (0.644–0.678)	0.550 (0.532–0.568)	2.39 (2.25–2.53)	<0.001	1.59 (1.48–1.72)	<0.001
LNR2	0.5<LNR	487 (2.9)	0.771 (0.734–0.810)	0.446 (0.402–0.494)	0.315 (0.275–0.362) ^a^	4.68 (4.19–5.23)	<0.001	3.17 (2.80–3.59)	<0.001
LODDS									
LODDS1	LODDS ≤ − 0.68	13,649 (82.4)	0.945 (0.941–0.949)	0.848 (0.842–0.854)	0.778 (0.770–0.785)	Reference		Reference	
LODDS2	− 0.68 < LODDS	2925 (17.6)	0.857 (0.844–0.870)	0.642 (0.624–0.660)	0.533 (0.515–0.553)	2.33 (2.19–2.47)	<0.001	1.70 (1.59–1.82)	<0.001
N-NPLN									
N-NPLN0	N0-NPLN0	12,927 (78.0)	0.950 (0.946–0.954)	0.862 (0.856–0.868)	0.795 (0.788–0.803)	Reference		Reference	
N-NPLN1	N1-NPLN1	2054 (12.4)	0.876 (0.861–0.890)	0.683 (0.663–0.704)	0.572 (0.550–0.595)	2.25 (2.09–2.41)	<0.001	1.53 (1.41–1.66)	<0.001
N-NPLN2	N1-NPLN2& N2-NPLN1	1180 (7.1)	0.841 (0.820–0.862)	0.593 (0.565–0.623)	0.488 (0.459–0.518)	2.86 (2.64–3.11)	<0.001	1.94 (1.76–2.14)	<0.001
N-NPLN3	N2-NPLN1	413 (2.5)	0.799 (0.761–0.839)	0.493 (0.446–0.545)	0.348 (0.303–0.399) ^a^	4.18 (3.70–4.71)	<0.001	2.65 (2.31–3.04)	<0.001
N-LNR									
N-LNR0	N0-LNR0	12,927 (78.0)	0.950 (0.946–0.954)	0.862 (0.856–0.868)	0.795 (0.788–0.803)	Reference		Reference	
N-LNR1	N1-LNR1	2111 (12.7)	0.875 (0.861–0.890)	0.682 (0.661–0.702)	0.574 (0.553–0.596)	2.22 (2.07–2.39)	<0.001	1.50 (1.38–1.64)	<0.001
N-LNR2	N1-LNR2	1049 (6.3)	0.856 (0.835–0.877)	0.620 (0.590–0.651)	0.502 (0.472–0.535)	2.73 (2.50–2.99)	<0.001	1.83 (1.65–2.04)	<0.001
N-LNR3	N2-LNR1	138 (0.8)	0.823 (0.761–0.890)	0.527 (0.448–0.620)	0.389 (0.312–0.486) ^b^	3.85 (3.12–4.74)	<0.001	2.77 (2.24–3.44)	<0.001
N-LNR4	N2-LNR2	349 (2.1)	0.750 (0.706–0.798)	0.414 (0.363–0.471)	0.286 (0.240–0.340) ^a^	5.07 (4.47–5.75)	<0.001	3.46 (3.00–4.00)	<0.001
N-LODDS									
N-LODDS0	N0-LODDS1	12,287 (74.1)	0.951 (0.947–0.955)	0.864 (0.858–0.871)	0.798 (0.791–0.805)	Reference		Reference	
N-LODDS1	N0-LODDS2	640(3.9)	0.933 (0.913–0.953)	0.817 (0.787–0.849)	0.744 (0.709–0.780)	1.30 (1.13–1.50)	<0.001	1.34 (1.16–1.55)	<0.001
N-LODDS2	N1-LODDS1	1059(6.4)	0.888 (0.869–0.908)	0.713 (0.685–0.741)	0.605 (0.575–0.636)	2.06 (1.87–2.26)	<0.001	1.33 (1.20–1.48)	<0.001
N-LODDS3	N1-LODDS2& N2-LODDS1	1493(9.0)	0.864 (0.847–0.882)	0.639 (0.615–0.665)	0.531 (0.505–0.558)	2.57 (2.38–2.78)	<0.001	1.80 (1.64–1.97)	<0.001
N-LODDS4	N2-LODDS2	1095(6.6)	0.813 (0.790–0.837)	0.546 (0.517–0.577)	0.421 (0.391–0.452)	3.49 (3.21–3.79)	<0.001	2.38 (2.14–2.64)	<0.001

Survival rate calculated using the Kaplan–Meier method

^a^5 year survival rate due to no fatal events at 60 months using 59 months of data

^b^5 year survival rate due to no fatal events at 60 months using 57 months of data

Multivariate analysis was adjusted for sex, age group, histology, tumor size, differentiation grade, tumor extension, radiotherapy, and systemic therapy

**Fig. 2:**
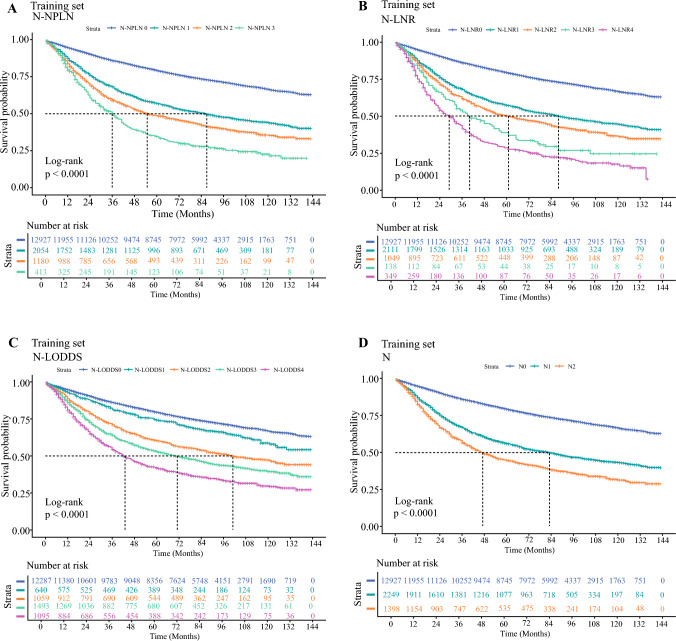

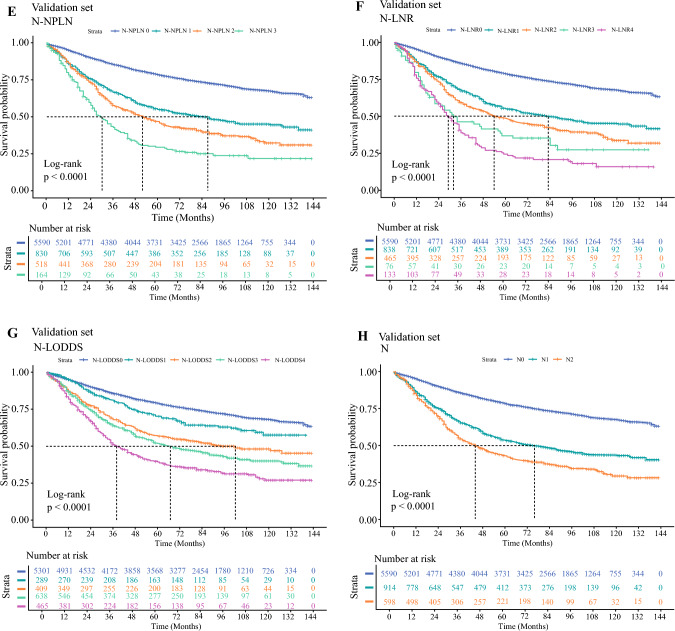
Kaplan–Meier curves for cancer-specific survival in patients with non-small-cell lung cancer: N-NPLN (**A** and **E**), N-LNR (**B** and **F**), N-LODDS (**C** and **G**), and N Stage (**D** and **H**) in training and validation sets

Groups showing minimal survival differences and nonsignificant pairwise comparisons (N1-NPLN2 vs. N2-NPLN1, *P*=0.634; N1-LODDS2 vs. N2-LODDS1, *P*=0.546) were subsequently consolidated, ultimately yielding four-, five-, and five-tier hierarchical systems for N-NPLN, N-LNR, and N-LODDS, respectively (Supplementary Figure 1 and Supplementary Figure 2). Univariate and multivariate Cox regression confirmed all three variables as independent prognostic factors (*P*<0.001) (Table 2). Time-dependent ROC analysis (Supplementary Figure 3) demonstrated that N-NPLN, N-LNR, N-LODDS, and conventional N staging achieved area under the curve (AUC) values for 1, 3, and 5 year prognostic predictions. Notably, N-LODDS exhibited superior discriminative performance, with the highest AUC values (1 year: 0.633; 95% CI 0.617–0.649; 3 year: 0.639; 95% CI 0.629–0.649; 5 year: 0.631; 95% CI 0.622–0.640), along with broad survival curve stratification. Log-rank tests demonstrated statistically significant inter-subgroup differences, with 5 year CSS rates differing by 5.4–13.9% between adjacent groups, indicating marked intergroup heterogeneity (Fig. 2 and Table 2).

### Development of the Nomogram

**Fig. 3:**
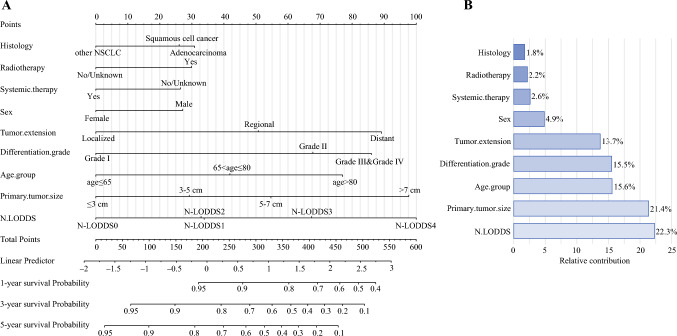
Nomogram for predicting 1, 3, and 5 year cancer-specific survival in patients with non-small-cell lung cancer (**A**) and variable importances on cancer-specific survival prediction (**B**)

Univariate Cox regression analysis in the training cohort revealed significant associations between CSS and N-LODDS, sex, age group, histology, tumor size, tumor extension, differentiation grade, radiotherapy, and systemic therapy (Supplementary Table 1, *P*<0.05). Multivariate analysis identified these nine variables as independent prognostic factors: N-LODDS (N-LODDS1: hazard ratio [HR] 1.34; 95% CI 1.16–1.55; N-LODDS2: HR 1.33; 95% CI 1.20–1.48; N-LODDS3: HR 1.80; 95% CI 1.64–1.97; N-LODDS4: HR 2.38; 95% CI 2.14–2.64), sex (male: HR 1.27; 95% CI 1.20–1.34), age group (>65–80 years: HR 1.44; 95% CI 1.36–1.5; >80 years: HR 1.95; 95% CI 1.76–2.17), histology (squamous cell carcinoma: HR 1.30; 95% CI 1.23–1.38; other NSCLC: HR 0.71; 95% CI 0.65–0.79), tumor size (3–5 cm: HR 1.29; 95% CI 1.21–1.38; 5–7 cm: HR 1.61; 95% CI 1.47–1.76; >7 cm: HR 2.33; 95% CI 2.11–2.58), tumor extension (localized: HR 1.55; 95% CI 1.45–1.67; distant: HR 2.17; 95% CI 1.89–2.50), differentiation grade (grade II: HR 1.80; 95% CI 1.64–1.98; grade Ⅲ and grade Ⅳ: HR 2.11; 95% CI 1.92–2.33), radiotherapy (yes: HR 1.30; 95% CI 1.18–1.42), and systemic therapy (yes: HR 0.79; 95% CI 0.74–0.86). A nomogram incorporating these predictors was developed for 1, 3, and 5 year CSS prediction (Fig. 3). The line lengths in the nomogram reflect variable contributions, with N-LODDS staging (22.3%) and tumor size (21.4%) demonstrating the highest relative importance, followed by age group (15.6%), differentiation grade (15.5%), and tumor extension (13.7%). Sex, histology, radiotherapy, and comprehensive treatment showed comparatively smaller predictive impacts.

### Validation and Comparison of Nomogram Performance

**Fig. 4:**
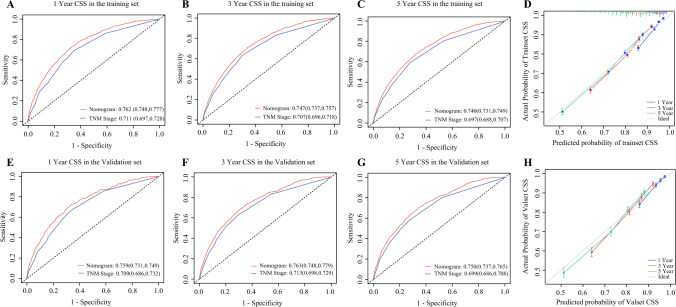
Receiver operating characteristic curves (**A**-**C**, **E**-**G**) and calibration plots (**D**, **H**) for 1, 3, and 5 year lung cancer-specific survival (CSS) prediction by nomogram, TNM and Surveillance, Epidemiology, and End Results staging in non-small-cell lung cancer

**Fig. 5:**
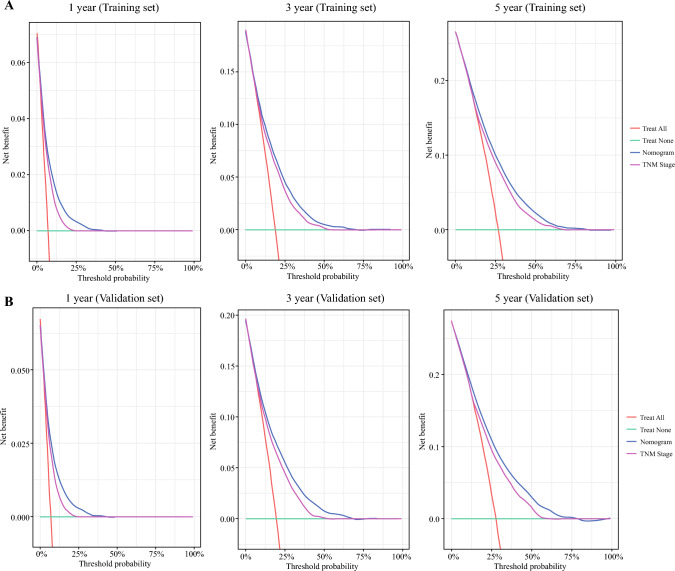
Decision curve analysis of nomogram, TNM staging, and Surveillance, Epidemiology, and End Results staging for predicting 1, 3, and 5 year cancer-specific survival in training (**A**) and validation sets (**B**)

The nomogram’s performance was systematically evaluated using training and validation cohorts. Time-dependent ROC analysis demonstrated superior discriminatory power, with AUC values of 0.762 (95% CI 0.748–0.777), 0.747 (95% CI 0.37–0.757), and 0.740 (95% CI 0.731–0.749) for 1, 3, and 5 year CSS, in the training set, respectively (Fig. 4A-C). The results were consistently replicated in the validation cohort and were significantly better than with TNM stage (Fig. 4E-G). Calibration curves confirmed excellent agreement between nomogram-predicted and observed survival rates in both cohorts (Fig. 4D,H). Decision curve analysis revealed enhanced clinical net benefit across wide threshold probability ranges compared with conventional TNM stage (Fig. 5). Furthermore, significant improvements in net reclassification improvement and integrated discrimination improvement were achieved for all CSS predictions (all* P*<0.001, Supplementary Table 2). Collectively, this nomogram surpasses existing staging systems in discriminative accuracy, calibration capability, and clinical utility, providing a robust tool for personalized prognostic assessment.

## Discussion

This study systematically evaluated the prognostic predictive efficacy of three lymph node metastasis quantification variables integrated with conventional N staging, utilizing clinicopathological characteristics and follow-up data from 23,676 patients with NSCLC in the SEER database (2010–2015).

Through comparative analysis, N-LODDS was identified as the optimal prognostic stratification variable. A prognostic nomogram for predicting CSS was subsequently developed based on this novel N-LODDS staging system. Validation results demonstrated that the nomogram significantly outperformed the traditional TNM staging system in discriminative accuracy, calibration capability, and clinical utility, highlighting its reliability and superiority as an individualized prognostic assessment tool for optimizing clinical treatment decision-making. Furthermore, this research confirmed the significance of N-LODDS as an independent prognostic factor in NSCLC while revealing limitations in current International Association for the Study of Lung Cancer’s 8th/9th edition *N* staging systems regarding lymph node metastasis evaluation and prognostic prediction, providing novel insights and evidence for refining future lung cancer staging frameworks.

This study excluded pN0 patients when determining optimal cutoff values for NPLN and LNR, as both variables equal zero in pN0 cases and demonstrate no discriminative capacity within this subgroup. The results established four positive lymph nodes as the optimal NPLN cutoff value, consistent with the documented three to four node range,^18,23–25^ whereas the LNR threshold of 0.5 aligned with the previously reported 21–50% interval.^18,23,25^ These findings collectively validate NPLN and LNR as independent prognostic factors. In contrast, the log odds of positive lymph nodes (LODDS) retained pN0 cases in its cutoff analysis through its mathematical formula that dynamically incorporates both positive lymph node count and total examined lymph nodes. This characteristic enables LODDS to generate differential values even in node-negative scenarios based on varying total lymph node examination numbers, thereby overcoming the inherent limitations of NPLN and LNR applications in pN0 staging.^26,27^ Previous studies have included NPLN, LNR, and LODDS as new N classifications alone^28–30^ or in combination with N staging^18^ or have combined NPLN or LNR with N staging as new N classifications.^23,31^ However, no study has systematically compared the prognostic predictive performance of different lymph node quantification variables when combined with *N* staging.

The results showed that the prognostic differentiation ability of N-LODDS was better than that of N-LNR and N-NPLN, with a wider distribution of Kaplan–Meier survival curves, which was able to make full use of the underlying lymph node characteristics and could effectively identify subgroups with poor prognosis in traditional N0 staging, providing the possibility of more reliable N classification. Log-rank testing revealed no statistically significant survival difference between the N1-LODDS2 and N2-LODDS1, leading to their consolidation into the N-LODDS3. This finding suggests that inadequate lymph node evaluation in node-positive patients may cause staging downgrades (e.g., misclassifying true N2 cases as N1), thereby affecting therapeutic decision-making. The existing studies also support that adequate lymph node dissection, which achieves precise staging by obtaining a sufficient number of lymph nodes, can result in a significant survival benefit for patients.^32^

The subdivision of N0 staging into N-LODDS1 and N-LODDS2 using LODDS variables demonstrated significantly worse survival outcomes for N-LODDS2 than for N-LODDS1 in both univariate and multivariate Cox regression analyses. These findings suggest that insufficient lymph node dissection or evaluation remains a prognostic risk factor, even in clinically node-negative cases, implying the N0 category may include occult cases with actual N1/N2 metastases. It has been demonstrated that a higher number of lymph nodes examined with more accurate lymph node staging correlates with long-term survival in patients with NSCLC.^33^ Notably, the multifactorial Cox model adjusted for covariates resulted in similar HR values for N1-LODDS1 and N0-LODDS2, possibly reflecting improved survival in patients with adequate lymph node dissection due to precise staging to receive appropriate adjuvant therapy, while re-validating the risk of lymph node metastasis leakage in N0 stage.

In this study, nine independent prognostic factors were screened by univariate and multivariate Cox regression analyses, of which N-LODDS was the most important factor in predicting CSS in patients with NSCLC. Compared with previous studies that mostly used T stage or tumor size as the core predictor, it may indicate that the combination of traditional N stage and LODDS can fully tap the information represented by lymph node metastasis, which improves its predictive ability.^18,34,35^

The nomogram analysis demonstrated that increased tumor size,^35,36^ higher differentiation grade,^37^ and greater tumor extension^34^ were all significant indicators of poor prognosis, which aligns with previous research findings. Demographic factors serve as significant prognostic predictors, with poorer outcomes in elderly patients potentially associated with enhanced immunosuppression, diminished tumor-killing capacity,^38^ and physiological functional decline. The survival advantage in female patients may be associated with sex hormone-mediated modulation of the tumor microenvironment and lower exposure to smoking.^39^ Regarding treatment, systemic therapy demonstrates significant protective effects, and this finding is consistent with several studies,^40–42^ whereas postoperative radiotherapy, although identified as a risk factor with support from some studies,^43–45^ remains controversial in its clinical utility.^46,47^

This study has several limitations. First, the retrospective design and exclusive use of the SEER database may introduce selection and information biases due to the lack of critical prognostic data (smoking history, comorbidities, family history, etc.). Caution is warranted when interpreting these findings, and multicenter prospective studies are needed to validate generalizability. Second, the failure of the SEER database to differentiate between intact lymph nodes and tissue fragments, as well as variations in recording specifications for lymph node stations across healthcare organizations, are factors that can affect the accuracy of the lymph node data and thus the precision of the analysis. In addition, the SEER database lacks detailed information on specific lymph node stations, so we were unable to differentiate between N2 patients with combined N1 lymph node metastases or to directly assess the prognostic impact of specific lymph node stations. To compensate for this limitation, the present study used anatomical *N*-stage as the basis for stratification, which was subsequently subdivided in combination with quantitative indicators. This approach ensures that even if the total number of positive lymph nodes is the same, the model can still effectively differentiate between patients with different anatomical risk tiers, such as N1 and N2, thus maximizing the use of anatomical location and quantity information in the available data. Third, the N-LODDS classification is only applicable to patients undergoing systematic lymphadenectomy without neoadjuvant therapy, as current methods cannot reliably assess nodal tumor burden in preoperative or inoperable cases. As cases receiving neoadjuvant therapy were excluded, this study naturally did not cover patients with clinically significant stage N2 or extensive N1. Therefore, our cohort mainly represents the direct surgery population with low lymph node load and may also provide a more precise prognostic stratification for patients who are suitable for direct surgery but have postoperative pathologically confirmed lymph node metastases.

This study also has notable strengths. The innovative integration of N-LODDS staging, systematically compared with N-NPLN and N-NLR, first demonstrated its superior prognostic stratification capacity in NSCLC. A robust nomogram incorporating N-LODDS was developed and internally validated using SEER’s large-scale population data. With complete survival records and sufficient follow-up duration, the model exhibited excellent predictive performance for CSS, highlighting its clinical translational potential.

## Conclusion

This study developed an N-LODDS stage system integrating anatomical location and quantitative characteristics of lymph nodes based on the SEER database and constructed the first prognostic nomogram. By dynamically integrating the number of positive and total examined lymph nodes, N-LODDS demonstrated superior prognostic stratification and discriminative ability compared with AJCC TNM staging, effectively identifying high-risk N0 patients with occult metastases and validating the independent prognostic value of N-LODDS. Future multicenter prospective studies are required to further validate the model.

## Supplementary Information

Below is the link to the electronic supplementary material.

Supplementary file1 (DOCX 1108 KB)

## Data Availability

The data for this study are available in a public, open repository and can be accessed in its entirety through the official data portal after authorization of the data use agreement (https://seer.cancer.gov/data-software/).
