# Supplementary material for: N-LODDS: A Novel Integrated Lymph Node Staging System Enhancing Prognostic Accuracy in Non-Small-Cell Lung Cancer
Source: Ann Surg Oncol. 2026 Jan 20;33(5):4242–55. doi: 10.1245/s10434-025-19005-x (PMC13083349; doi:10.1245/s10434-025-19005-x)
Supplement: Supplementary file 1 — Supplementary file1 (DOCX 1108 KB) [file 10434_2025_19005_MOESM1_ESM.docx]

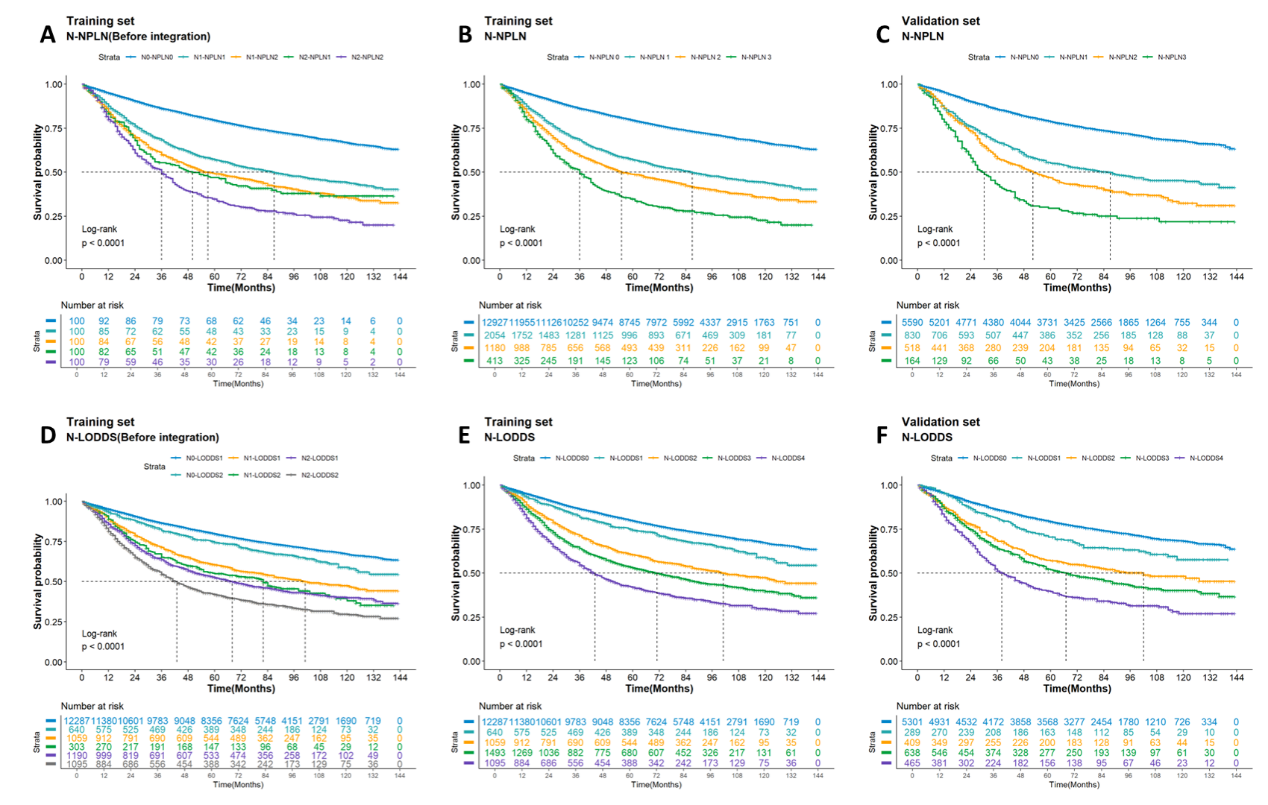


Supplementary Figure 1. Kaplan-Meier Curves of CCS in NSCLC: N-NPLN Staging Development (before integration, after integration, validation set) (A-C); N-LODDS Staging Development (before integration, after integration, validation set) (D-F)


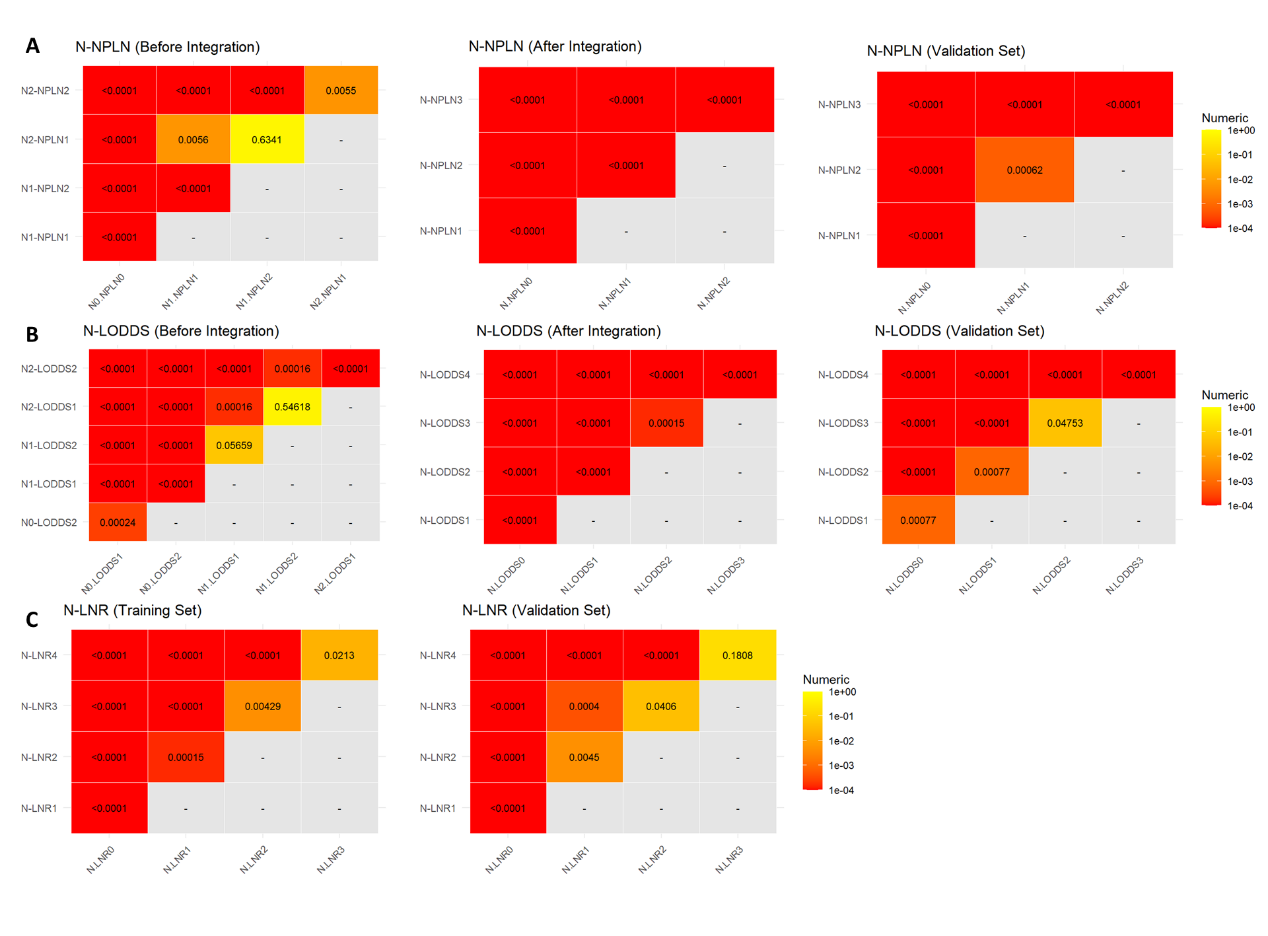


Supplementary Figure 2. Log-rank Test Results: Pairwise Comparisons Among Subgroups for N-NPLN, N-LODDS, and N-LNR Variables before and after integration


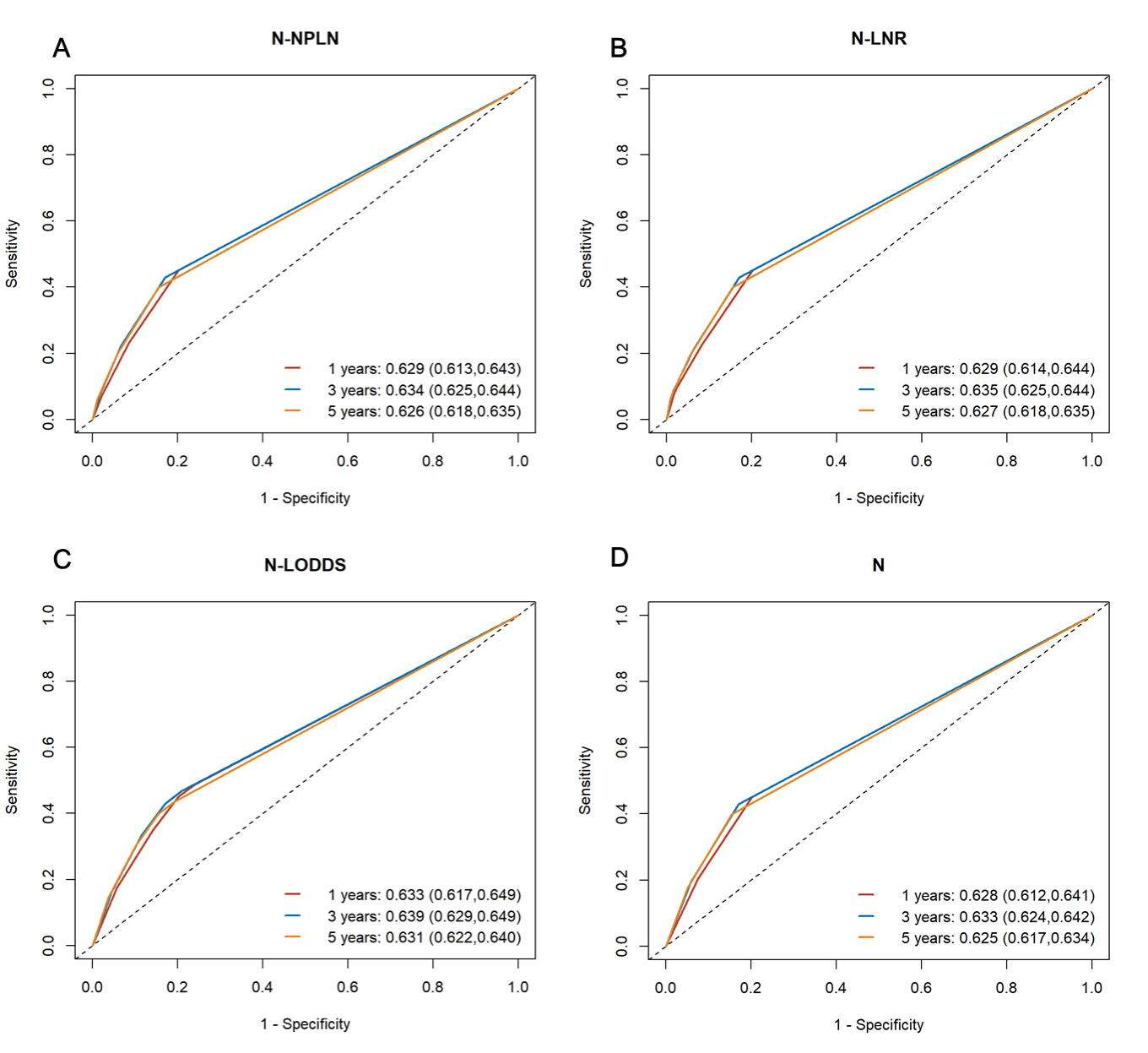


Supplementary Figure 3. ROC Curves for Predicting 1-, 3-, and 5-Year CCS in NSCLC Patients: N-NPLN (A), N-LNR (B), N-LODDS (C), and N-Stage (D).

Supplementary Table 1. Univariate and multivariate Cox regression analyses of prognostic factors in NSCLC patients

| **Variables** | **Univariate analysis** |  | **Multivariate analysis** | |
| --- | --- | --- | --- | --- |
|  | **HR (95% CI)** | ***P*** | **HR (95% CI)** | ***P*** |
| **Race** |  |  |  |  |
| White | Reference | |  |  |
| Black | 0.94 (0.85-1.03) | 0.194 |  |  |
| Other | 0.87 (0.79-0.96) | 0.008 |  |  |
| Unknown | 0.08 (0.01-0.59) | 0.013 |  |  |
| **Sex** |  |  |  |  |
| Female | Reference | | Reference | |
| Male | 1.40 (1.33-1.48) | <0.001 | 1.27 (1.20-1.34) | <0.001 |
| **Age group** |  |  |  |  |
| age≤65 | Reference | | Reference | |
| 65<age≤80 | 1.38 (1.31-1.46) | <0.001 | 1.44 (1.36-1.53) | <0.001 |
| 80<age | 1.87 (1.69-2.07) | <0.001 | 1.95 (1.76-2.17) | <0.001 |
| **Laterality** |  |  |  |  |
| Left | Reference | |  |  |
| Right | 0.98 (0.93-1.04) | 0.475 |  |  |
| **Primary site** |  |  |  |  |
| C34.1-Upper lobe, lung | Reference | |  |  |
| C34.2-Middle lobe, lung | 0.97 (0.86-1.09) | 0.586 |  |  |
| C34.3-Lower lobe, lung | 1.10 (1.03-1.16) | 0.002 |  |  |
| C34.0-Main bronchus | 0.84(0.60-1.17) | 0.297 |  |  |
| C34.8-Overlapping lesion of lung | 1.78 (1.47-2.16) | <0.001 |  |  |
| C34.9-Lung, NOS | 1.39 (1.11-1.73) | 0.004 |  |  |
| **Primary tumor size** |  |  |  |  |
| ≤3 cm | Reference | | Reference | |
| 3-5 cm | 1.67 (1.57-1.78) | <0.001 | 1.29 (1.21-1.38) | <0.001 |
| 5-7 cm | 2.35 (2.17-2.56) | <0.001 | 1.61 (1.47-1.76) | <0.001 |
| >7 cm | 3.17 (2.88-3.49) | <0.001 | 2.33 (2.11-2.58) | <0.001 |
| **Tumor extension** |  |  |  |  |
| Regional | Reference | | Reference | |
| Localized | 2.44 (2.31-2.58) | <0.001 | 1.55 (1.45-1.67) | <0.001 |
| Distant | 3.22 (2.81-3.68) | <0.001 | 2.17 (1.89-2.50) | <0.001 |
| **Histology** |  |  |  |  |
| Adenocarcinoma | Reference | | Reference | |
| Squamous cell cancer | 1.30 (1.23-1.38) | <0.001 | 0.96 (0.90-1.02) | 0.184 |
| other NSCLC | 0.71 (0.65-0.79) | <0.001 | 0.76 (0.69-0.85) | <0.001 |
| **Grade** |  |  |  |  |
| Grade I | Reference | | Reference | |
| Grade II | 2.33 (2.12-2.55) | <0.001 | 1.80 (1.64-1.98) | <0.001 |
| Grade III & Grade IV | 3.08 (2.80-3.38) | <0.001 | 2.11 (1.92-2.33) | <0.001 |
| **Systemic therapy** |  |  |  |  |
| No/unknown | Reference | | Reference | |
| Yes | 1.83 (1.73-1.93) | <0.001 | 0.79 (0.74-0.86) | <0.001 |
| **Radiotherapy** |  |  |  |  |
| No/unknown | Reference | | Reference | |
| Yes | 2.34 (2.17-2.54) | <0.001 | 1.30 (1.18-1.42) | <0.001 |
| **N-LODDS** |  |  |  |  |
| N-LODDS0 | Reference | | Reference | |
| N-LODDS1 | 1.30 (1.13-1.50) | <0.001 | 1.34 (1.16-1.55) | <0.001 |
| N-LODDS2 | 2.06 (1.87-2.26) | <0.001 | 1.33 (1.20-1.48) | <0.001 |
| N-LODDS3 | 2.57 (2.38-2.78) | <0.001 | 1.80 (1.64-1.97) | <0.001 |
| N-LODDS4 | 3.49 (3.21-3.79) | <0.001 | 2.38 (2.14-2.64) | <0.001 |

Supplementary Table 2. NRI and IDI Comparison for CSS Prediction in NSCLC: Nomogram vs. TNM Staging.

| **Index** | **Training set** | | **Validation set** | |
| --- | --- | --- | --- | --- |
|  | **Estimate (95%CI)** | ***P*** | **Estimate(95%CI)** | ***P*** |
| **NRI (vs. TNM stage)** |  |  |  |  |
| 1-year CSS | 0.383 (0.33-0.44) | <0.001 | 0.366 (0.277-0.455) | <0.001 |
| 3-year CSS | 0.236 (0.20-0.27) | <0.001 | 0.301 (0.242-0.359) | <0.001 |
| 5-year CSS | 0.233 (0.20-0.27) | <0.001 | 0.291 (0.239-0.343) | <0.001 |
| **IDI (vs. TNM stage)** |  |  |  |  |
| 1-year CSS | 0.023 (0.020-0.030) | <0.001 | 0.025 (0.020-0.030) | <0.001 |
| 3-year CSS | 0.031 (0.027-0.034) | <0.001 | 0.036 (0.031-0.042) | <0.001 |
| 5-year CSS | 0.033 (0.030-0.040) | <0.001 | 0.039 (0.033-0.044) | <0.001 |
